# Supplementary material for: Rescuing error control in crosslinking mass spectrometry
Source: Mol Syst Biol. 2024 Aug 2;20(9):1076–84. doi: 10.1038/s44320-024-00057-2 (PMC11368935; doi:10.1038/s44320-024-00057-2)
Supplement: Supplementary file 2 — Expanded View Figures [file 44320_2024_57_MOESM2_ESM.pdf]

Expanded View Figure

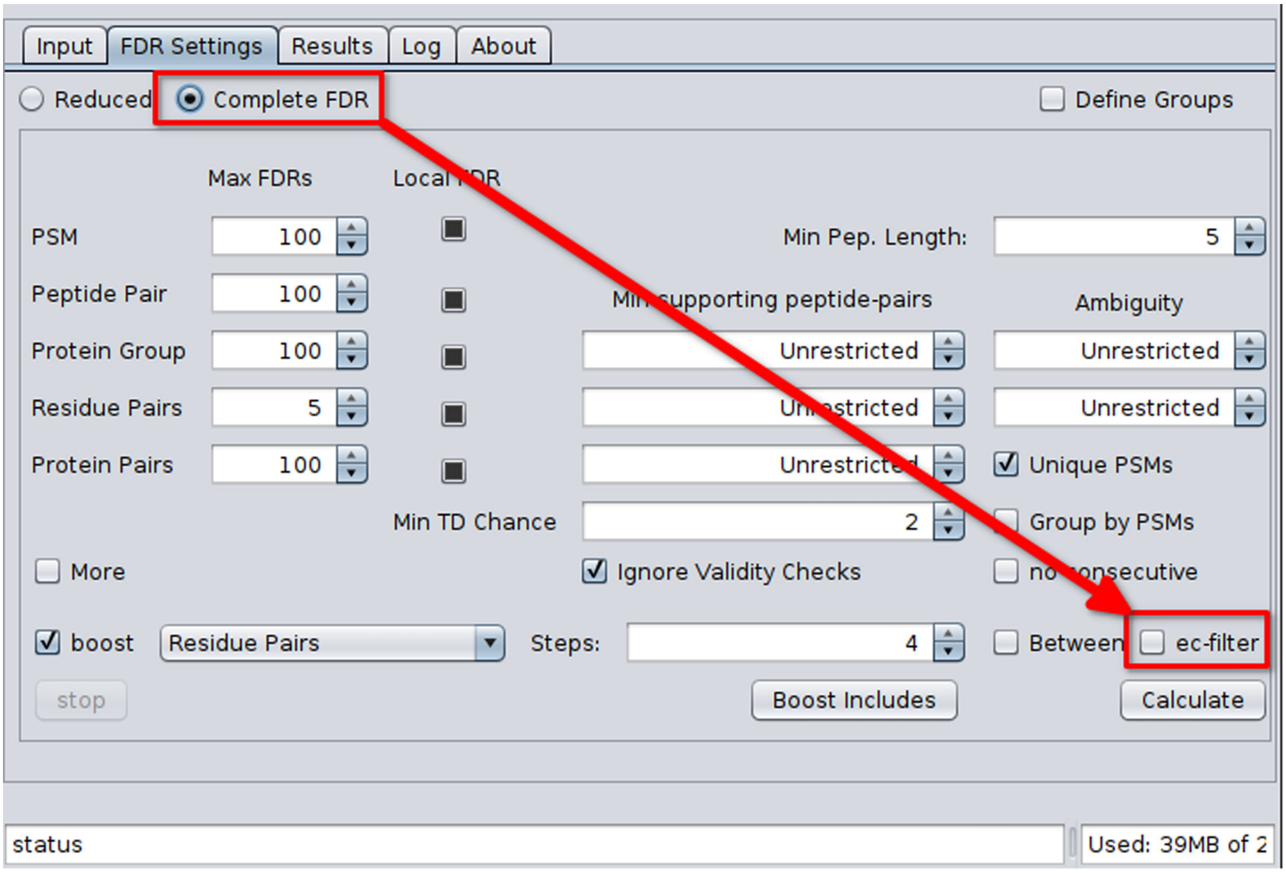

Figure EV1. xiFDR ec-filter selection.

To use the ec-filter the complete settings need to be used and the ec-filter checkbox ticked.
